# Modelling COVID 19 in the Basque Country from introduction to control measure response

**DOI:** 10.1038/s41598-020-74386-1

**Published:** 2020-10-14

**Authors:** Maíra Aguiar, Eduardo Millán Ortuondo, Joseba Bidaurrazaga Van-Dierdonck, Javier Mar, Nico Stollenwerk

**Affiliations:** 1 Dipartimento di Matematica, Università degli Studi di Trento, Via Sommarive, 14, 38123 Povo, Trento, Italy; 2 Basque Center for Applied Mathematics (BCAM), Alameda Mazarredo, 14, 48009 Bilbao, Spain; 3 Ikerbasque, Basque Foundation for Science, Bilbao, Spain; 4 Osakidetza Basque Health Service, General Sub-directorate for Healthcare, Vitoria-Gasteiz, Spain; 5 Public Health, Basque Health Department, Rekalde Zumarkalea 39A, 48008 Bilbao, Spain; 6 Research Unit, Osakidetza Basque Health Service, Debagoiena Integrated Healthcare Organisation, Arrasate-Mondragón, Guipúzcoa Spain; 7 Biodonostia Health Research Institute, Donostia-San Sebastián, Guipúzcoa Spain; 8 Economic Evaluation Unit, Kronikgune Institute for Health Services Research, Barakaldo, Spain; 9 Center for Mathematics, Fundamental Applications and Operations Research, Lisbon University, Lisbon, Portugal

**Keywords:** Computational biology and bioinformatics, Systems biology, Diseases

## Abstract

In March 2020, a multidisciplinary task force (so-called Basque Modelling Task Force, BMTF) was created to assist the Basque health managers and Government during the COVID-19 responses. BMTF is a modelling team, working on different approaches, including stochastic processes, statistical methods and artificial intelligence. Here we describe the efforts and challenges to develop a flexible modeling framework able to describe the dynamics observed for the tested positive cases, including the modelling development steps. The results obtained by a new stochastic SHARUCD model framework are presented. Our models differentiate mild and asymptomatic from severe infections prone to be hospitalized and were able to predict the course of the epidemic, providing important projections on the national health system’s necessities during the increased population demand on hospital admissions. Short and longer-term predictions were tested with good results adjusted to the available epidemiological data. We have shown that the partial lockdown measures were effective and enough to slow down disease transmission in the Basque Country. The growth rate $\lambda$ was calculated from the model and from the data and the implications for the reproduction ratio *r* are shown. The analysis of the growth rates from the data led to improved model versions describing after the exponential phase also the new information obtained during the phase of response to the control measures. This framework is now being used to monitor disease transmission while the country lockdown was gradually lifted, with insights to specific programs for a general policy of “social distancing” and home quarantining.

## Introduction

In December 2019, a new respiratory syndrome called coronavirus disease 2019 (COVID-19) caused by a new coronavirus (SARS-CoV-2)^1^ was identified in China^2^ and spread rapidly around the globe. With human-to-human transmission confirmed in 3 countries outside China, COVID-19 was declared a Public Health Emergency of International Concern by the World Health Organization (WHO) on 30 January 2020. By February 25, 2020, China was the epicenter of the outbreak and, in 2 weeks, on March 11, 2020, COVID-19 was characterized as a pandemic, with Europe reporting more cases and deaths than the rest of the world combined, apart from China^3^. Up to April 25, 2020, more than 2.8 million cases were confirmed with about 200 thousand deaths, with a global case fatality ratio (CFR) of approximate 7%^4^.

Italy, the first hardest hit country in Europe, had local transmission confirmed in all regions in the beginning of March, 2020. Eleven municipalities in northern Italy were identified as the centers of the two main Italian clusters. On March 8, 2020, the Prime Minister Giuseppe Conte had placed in quarantine all of Lombardy and 14 other northern provinces. The national lockdown decree was signed on March 9, 2020^5^, prohibiting all forms of gathering of people in public places and suspending sports events and competitions of all types. By that time, Italy, was considered the new epicenter of the outbreak, reporting more than 9 thousand confirmed cases with more than 450 deaths. On March 21, 2020, further restrictions within the nationwide lockdown were imposed with all non-essential production, industries and businesses halted, as the number of new cases and deaths were still rising. In respect to the total number of confirmed cases, Spain was 8 days behind Italy, with cases reported in all 50 provinces of the country on March 8, 2020. The decree of a national lockdown was signed on March 14, 2020^6^, with all non-essential workers staying at home from March 27, 2020, onwards^7,8^.

In the Basque Country, an autonomous community in northern Spain with 2.2 milion inhabitants, the first cases of COVID-19 were notified on March 4, 2020. A public health emergency was declared before any other region in Spain^9^. All schools in the Basque Country were closed by March 12, 2020, and, ruled by the same Spanish decrees^6–8^, lockdown measures were implemented accordingly and in time. An extension of the state of alarm was published on April 10, 2020^10^, and although teleworking is still prioritized, some restrictions started to be lifted, with workers in some non-essential sectors allowed to return to work using face masks on April 13, 2020, and children under the age of 14 allowed to go outside for a walk, within a one-kilometer radius of their home, on April 26, 2020^11^. The national plan for lifting the restrictions imposed during the state of alarm called “Plan for the Transition towards a new normality” was announced on April 28, 2020^12^, and will take place over 4 phases with a “gradual, flexible and adaptive” de-escalation to “a new normality”, depending on the on-going progress of COVID-19 epidemic’s control across the different regions of Spain. Started on May 4, 2020, with its “Phase Zero”, the proposed plan will last eight weeks, until the end of June.

As the COVID-19 pandemic is unfolding, research on mathematical modelling becomes more important than ever to understand disease spreading dynamics and the impact of intervention measures. By incorporating the new information generated by virology, field epidemiology and social behaviour, for example, mathematical models are often used to guide public health authorities with projections for the national health system’s necessities during an outbreak. Those mathematical models also provide insights about the disease spreading over time, assessing the impact of human interventions for disease control, and Governments in some countries have already taking important decisions based on these results^13–15^. Worldwide country lockdowns are unprecedented extreme measures recently taken and, although needed to decelerate disease transmission, have caused a huge economic crises around the globe. As some countries on the northern hemisphere start to announce that they were able to control the spreading of the disease and have now “reached the peak” of the epidemic, Governments start to consider to relax the imposed restrictions, and once again, mathematical models become essential guiding tools to evaluate the impact of the ongoing partial lockdowns lifting on disease transmission intensities, for different epidemiological scenarios, combined with many other public health measures that must take place for continuing COVID-19 prevention and mitigation such as testing, contact tracing and isolation of infected individuals.

The COVID-19 pandemic has resulted in an avalanche of epidemiological modelling papers, most of them using simple models such as the SIR (Susceptible-Infected- Recovered) or SEIR (Susceptible-Exposed-Infected- Recovered) in mechanistic or probabilistic frameworks to understand and predict the spread of the disease in a population. Modelling the dynamics of COVID-19 is very challenging, as we know very little about disease transmission. More complex models would be able to give more accurate projections about specific variables such as number of hospitalizations, intensive care units admissions (ICUs) and deaths, for example, over the course of the epidemics. However, to build useful models, good quality empirical data and its understanding, as well as a close collaboration among mathematical modelers, field and laboratory researchers as well as public health stakeholders are essential^16–21^.

In March 2020, a multidisciplinary task force (so-called Basque Modelling Task Force, BMTF) was created to assist the Basque Health managers and the Basque Government during the COVID-19 responses. BMTF is a modelling team, working on different approaches, including stochastic processes, statistical methods and artificial intelligence. Members were collaborating taking into consideration all information provided by the public health frontline and using different available datasets in respect to the COVID-19 outbreak in the Basque Country. The primary objectives were: (i) projections on the national health system’s necessities during the increased population demand on hospital admissions while the epidemic was unfolding; (ii) description of the epidemic in terms of disease spreading and control and (iii) monitoring the disease transmission when the country’s lockdown was gradually lifted. All modelling approaches were complementary and were able to provide coherent results, assuring that the decisions made using the modelling results were sound and, in fact, adjusted to the current epidemiological data. In addition, modelling results provided useful predictive information to validate outbreak control decisions and finally, to assist authorities in the Basque Country.

We use stochastic SHARUCD-type models (susceptible (S), severe cases prone to hospitalization (H), mild, sub-clinical or asymptomatic (A), recovered (R), patients admitted to the intensive care units (U) and the recorded cumulative positive cases (C) which includes all new positive cases for each class of H, A, U, R, and deceased (D))—an extension of the well known simple SIR model that is frequently used to model different disease outbreaks^28–30^. The deterministic approach is obtained via the mean field approximation of the stochastic system and is also used to evaluate the model performance and accuracy to guide the modeling analysis. The model is calibrated using the empirical data for the Basque Country community and the biological parameters are either estimated or fixed as the model is able to describe the disease incidence data. The growth rate ($\lambda$) was calculated from the model and from the data and results for the reproduction ratio (*r*) are shown. As the epidemic has entered into its linear phase, with the new number of cases increasing less and stabilizing, we keep monitoring, within the BMTF, the effect of the control measures on disease transmission. As this paper is revised, it is important to mention that the longer-term predictions of the development of the epidemic in the Basque Country turned out to be correct, with the notified cases well within the $95\%$ confidence intervals for the studied epidemiological scenario (from introduction toward control).

In this paper we describe our efforts as a task force to develop a flexible modeling framework, able to describe the dynamics observed for the tested positive cases, hospitalizations, intensive care units (ICUs) admissions, deceased and finally the recovered. Keeping the biological parameters for COVID-19 in the range of the recent research findings^22–27^, but adjusting to the phenomenological data description, we were able to explain well the exponential phase of the epidemic and later to evaluate the effect of the imposed control measures. While using the real time data provided by the Basque Health Managers, from March 4 up to May 4, we were able to refine the models as we learned from the epidemic in the Basque Country. As a follow up exercise, while the epidemic was unfolding and the control measures were implemented, our results are presented and discussed. These are the first publicly available modelling results for the Basque Country and the efforts are continued taking into consideration the updated data and new information that are generated over time. Preparation for a possible second wave of transmission is also discussed, but not explicitly modeled, as the eventual influence of seasonality and the role of acquired immunity are not clear nor well measurable yet.

## Methods and results

### Epidemiological data and data inspection

**Figure 1:**
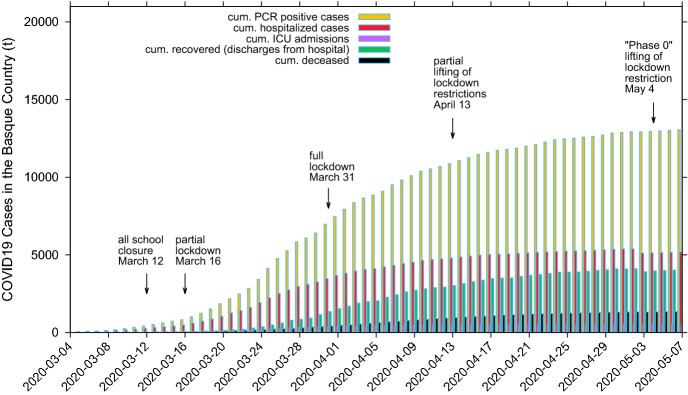
Cumulative COVID-19 cases of tested positive ($I_{cum}$), hospitalized cases ($C_H$), ICU admission ($C_U$), recovered ($C_R$) and deceased cases (*D*). The imposed control measures and its gradual lifting process are marked with arrows with the effective dates of implementation.

Epidemiological data used in this study are provided by the Basque Health Department and the Basque Health Service (Osakidetza), continually collected with specific inclusion and exclusion criteria, and for the present analysis, the last update was on May 4, 2020. This is a dynamical work and new results are presented throughout the manuscript as new data are collected to calibrate the models. We use the following incidence and cumulative data for RT-PCR (reverse transcriptase-polymerase chain reaction) tested positive patients (yellow), recorded as hospital admissions (red), intensive care units admissions (purple), recovered (green) and deceased (black), as shown in Fig. 1. The remaining patients are assumed to be individuals with milder infections. Data sets for hospitalizations, ICU admissions, discharges and deceases were obtained from the Osakidetza’s Business Intelligence (OBI) platform, enabling us to exploit and analyze the empirical data from the main information systems of Basque Health Service, including the structured data from the electronic health records of the Basque Health Service. In a different format, data is publicly available and can be retrieved from^31,32^.

At the beginning of the outbreak, RT-PCR tests were only performed to those patients with severe symptoms admitted to hospital. From March 22 onwards, testing capacities have increased, with antibody tests introduced on April 6, 2020, and used mainly as screening tool in nursing homes, where less severe symptomatic cases started to be tested. Data from different sources are linked by a pseudonymous identification of the patient.

#### Definition of each variable and data flow path withing the BMTF

Positives cases are patients tested positive for the first time with a RT-PCR test. Hospitalizations and ICUs refer to tested positive patients admitted to a hospital and intensive care unit respectively. A unique episode (hospitalization) is considered in case of patient transfer between hospitals in the Basque Country. To avoid including hospitalizations not related to COVID-19 infection, hospitalizations with a discharge date before the notification of the positive test are excluded and only hospitalizations that at a certain moment were in charge of services a priori responsible of attending COVID-19 disease are included: (internal medicine, pediatrics, ICU or reanimation services, respiratory services and infection disease services). The recovered variable refers to notified hospital and ICU discharges, excluding death. This data set does not include recovered individuals that were tested positive but not admitted to a hospital and like that represents only part of the expected recoveries in the population. As postmortem examination is not used as a tool of investigation prior to death notification^33^, deceased include both hospital and outside hospital deaths with positive PCR test.

To validate those variable definitions with the available data, the following path was used: (i) data are extracted from OBI and transformed following the above described definitions, (ii) data sets are shared with collaborators from different health organizations to compare and check the data collection and to eventually revise variable definitions, adjusting if necessary, (iii) data are used to validate mathematical models developed to describe COVID-19 dynamics in the Basque Country and results are discussed during regular BMTF meetings with regular reports delivered to the Basque Health managers and the Basque Government, (iv) feedback on the reports are sent to the researchers working with data with adjustments on models, parameters and data collection when necessary.

At first data inspection, the dynamics of the cumulative cases together with the effective starting dates for different control measures imposed are shown in Fig. 1.

### Modelling framework

We use SHARUCD-type models, an extension of the well known simple *SIR* (susceptible-infected-recovered) model, with infected class *I* partitioned into severe infections prone to hospitalization (*H*) and mild, sub-clinical or asymptomatic infections (*A*). From a typical SIR model with constant population size $N=S+I+R$, infection rate $\beta$ and recovery rate $\gamma$ (and an eventual waining immunity rate $\alpha$, which in the case of COVID-19 is not yet relevant as we assume, preliminarily, that infection leads to immunity during the time horizon considered up to now) given by

1 $$\begin{aligned} \frac{d}{dt} S &= \alpha R -\beta \frac{S}{N} I \nonumber \\ \frac{d}{dt} I &=  \beta \frac{S}{N} I -\gamma I \nonumber \\ \frac{d}{dt} R &=  \gamma I -\alpha R \end{aligned}$$

we develop a basic SHAR-model including a severity ratio $\eta$ for susceptible individuals *S* developing severe disease and possibly being hospitalized *H*. Milder disease with ratio $(1-\eta )$, including sub-clinical and eventually asymptomatic infections *A*, is assumed to have different infectivity $\phi$ from severe hospitalized disease $\beta$. Here, the ratio $\phi$ could be smaller or larger than 1 compared to the baseline infectivity rate $\beta$, leading to an altered infectivity rate $\phi \cdot \beta$ for the *A* class.

The SHAR-type model^34^ is presented by the Eq. system 2, where parameters for disease induced death ($\mu$) and intensive care units admission ($\nu$) are indicated, being relevant for severe disease prone to hospitalization but not for mild/asymptomatic cases

2 $$\begin{aligned} \frac{d}{dt} S &=  -\beta \frac{S}{N} (H+\phi A) \nonumber \\ \frac{d}{dt} H &=  \eta \beta \frac{S}{N} (H+\phi A)-(\gamma + \mu + \nu )H \nonumber \\ \frac{d}{dt} A &=  (1-\eta )\beta \frac{S}{N} (H+\phi A)-\gamma A \nonumber \\ \frac{d}{dt} R &=  \gamma (H+A) \quad . \end{aligned}$$

This model sketch needs to be further refined adjusting the modelling framework to the available COVID-19 empirical data. For investigation of $\phi$ to be around one or even larger due to higher mobility of mild infected or asymptomatic see e.g.^35^.

For severe infections prone to hospitalization, we assume the following dynamics: severe hospitalized individuals *H* could either recover, with a recovery rate $\gamma$, be admitted to the ICU facilities *U*, with a rate $\nu$, or eventually, before being admitted to the ICU facilities, decease into class *D*, with a disease induced death rate $\mu$. The ICU admitted patients could recover or die. For completeness of the system and to be able to describe the initial phase of the epidemic, an import term $\varrho$ should be also included into the force of infection. The import term refers to the imported disease cases, either by infected travelers coming from abroad to the Basque Country and transmitting the disease before returning to their home country, or to Basque citizens becoming infected while abroad and returning to the Basque Country during their infectious period. This factor becomes important when the lockdown is completely lifted, allowing international traveling, but turned out not to be important in the initial exponential phase of the epidemic. For the present study, we assume $\varrho$ to be much smaller than the other additive terms of the force of infection, given the strong observational insecurities on the data collected at the beginning of the outbreak, when $\varrho$ would matter most.

As we investigate cumulative data on the infection classes and not prevalence, we also include classes *C* to count cumulatively the new cases for “hospitalized” $$C_H$$, “asymptomatic” $$C_A$$, recovered $$C_R$$ and ICU patients $$C_U $$. In this way we can easily include a ratio $$\xi $$ of under-notification of mild/asymptomatic cases. The deceased cases are automatically collecting cumulative cases, since there is no exit transition form the death class *D*. While *H* and *U* can describe the empirical data reported for each class, the recovered *R*, which are also cumulative, count all biologically recovered, including the undetected mild/asymptomatic cases. Therefore, the $$C_R $$ class is needed to describe the present data which count only the notified asymptomatic in terms of $$\xi A $$. We have finally the SHARUCD-type models, shown by Eq. system 3, where individual transitions are still subject to refinement upon information obtained about COVID-19 biological mechanisms and on additional information directly obtained from the data. For a basic first SHARUCD model we keep a balance between biologically necessary and relevant model classes, and transitions, and a possibly relatively low number of free parameters, able to be estimated with the presently available data, avoiding over-parametrization as much as possible.

The deterministic version of the model is given by a differential equation system for all classes, including the recording classes of cumulative cases $$C_H $$, $$C_A $$, $$C_R $$ and $$C_U $$ as

3 $$\begin{aligned} \frac{d}{dt} S &=  -\beta \frac{S}{N} (H+\phi A+\varrho N) \nonumber \\ \frac{d}{dt} H &=  \eta \beta \frac{S}{N} (H+\phi A+\varrho N)-(\gamma + \mu + \nu )H \nonumber \\ \frac{d}{dt} A &=  (1-\eta )\beta \frac{S}{N} (H+\phi A+\varrho N)-\gamma A \nonumber \\ \frac{d}{dt} R &=  \gamma (H+U+A) \nonumber \\ \frac{d}{dt} U &=  \nu \eta \beta \frac{S}{N} (H+\phi A+\varrho N)- (\gamma +\mu ) U \nonumber \\ \frac{d}{dt} C_H &=  \eta \beta \frac{S}{N} (H+\phi A+\varrho N) \nonumber \\ \frac{d}{dt} C_A &=  \xi \cdot (1-\eta ) \beta \frac{S}{N} (H+\phi A+\varrho N) \nonumber \\ \frac{d}{dt} C_R &=  \gamma (H+U+\xi A) \nonumber \\ \frac{d}{dt} C_U &=  \nu H \nonumber \\ \frac{d}{dt} D &=  \mu (H+U) \quad . \end{aligned}$$

We consider primarily SHARUCD model versions as stochastic processes in order to compare with the available data which are often noisy and to include population fluctuations, since at times we have relatively low numbers of infected in the various classes. The stochastic version can be formulated through the master equation^36–38^ in application to epidemiology^39,40^ in a generic form using densities of all variables $$x_1:=S/N $$, $$x_2:=H/N $$, $$x_3:=A/N $$, $$x_4:=R/N $$, $$x_5:=U/N $$, $$x_6:=C_H/N $$, $$x_7:=C_A/N $$, $$x_8:=C_U/N $$ and $$x_9:=D/N $$ and $$x_{10}:=C_R/N $$ hence state vector $${\underline{x}} := (x_1,\ldots ,x_{10})^{tr} $$, giving the dynamics for the probabilities $$p({\underline{x}},t) $$ as

4 $$\begin{aligned} \frac{d}{dt} \; p({\underline{x}},t) &=  \sum _{j=1}^{n} \left( \frac{}{} N w_j ({\underline{x}} + \Delta {\underline{x}}_j) \cdot p ({\underline{x}} + \Delta {\underline{x}}_j,t) \right. \left. \frac{}{} - N w_j ({\underline{x}} ) \cdot p({\underline{x}} ,t) \right) \end{aligned}$$

with $n=10$ different transitions $w_j ({\underline{x}})$, as described by the mechanisms above, and small deviation from state ${\underline{x}}$ as $\Delta {\underline{x}} _j := \frac{1}{N} \cdot {\underline{r}}_j$^40–42^. For the basic SHARUCD model we have explicitly the following transitions $w_j ({\underline{x}})$ and its shifting vectors ${\underline{r}}_j$ given by

$$\begin{aligned} \begin{array}{ll} w_1 ({\underline{x}}) = \eta \beta x_1 (x_2+\phi x_3+\varrho ), &{} {\underline{r}}_1 = (1,-1,0,0,0,-1,0,0,0,0)^{tr} \\ w_2 ({\underline{x}}) = \xi (1-\eta ) \beta x_1 (x_2+\phi x_3+\varrho ) , &{} {\underline{r}}_2 = (1,0,-1,0,0,0,-1,0,0,0)^{tr} \\ w_3 ({\underline{x}}) = (1-\xi ) (1-\eta ) \beta x_1 (x_2+\phi x_3+\varrho ) , &{} {\underline{r}}_3 = (1,0,-1,0,0,0,0,0,0,0)^{tr} \\ w_4 ({\underline{x}}) = \gamma x_2 , &{} {\underline{r}}_4 = (0,1,0,-1,0,0,0,0,0,-1)^{tr} \\ w_5 ({\underline{x}}) = (1-\xi ) \gamma x_3 , &{} {\underline{r}}_5 = (0,0,1,-1,0,0,0,0,0,0)^{tr} \\ w_6 ({\underline{x}}) = \gamma x_5 , &{} {\underline{r}}_6 = (0,0,0,-1,1,0,0,0,0,-1)^{tr} \\ w_7 ({\underline{x}}) = \nu x_2 , &{} {\underline{r}}_7 = (0,1,0,0,-1,0,0,-1,0,0)^{tr} \\ w_8 ({\underline{x}}) = \mu x_2 , &{} {\underline{r}}_8 = (0,1,0,0,0,0,0,0,-1,0)^{tr} \\ w_9 ({\underline{x}}) = \mu x_5 , &{} {\underline{r}}_9 = (0,0,0,0,1,0,0,0,-1,0)^{tr}\\ w_{10} ({\underline{x}}) = \xi \gamma x_3 , &{} {\underline{r}}_{10} = (0,0,1,-1,0,0,0,0,0,0,-1)^{tr} \quad . \end{array} \end{aligned}$$

With these $w_j ({\underline{x}})$ and ${\underline{r}}_j$ specified we also can express the mean field ODE system as shown in the supplementary material giving as result the Eq. system 3.

### Model simulations and data

Considering as starting points the biological aspects of the disease^26,27,43–46^, the model is calibrated and parametrized using the cumulative empirical COVID-19 incidence data for $C_H$, $C_U$ and *D* directly on the respective data sets, and for the positive tested infected $I_{cum}$, which includes the new reported cases in all recorded classes, except the recovered.

**Figure 2:**
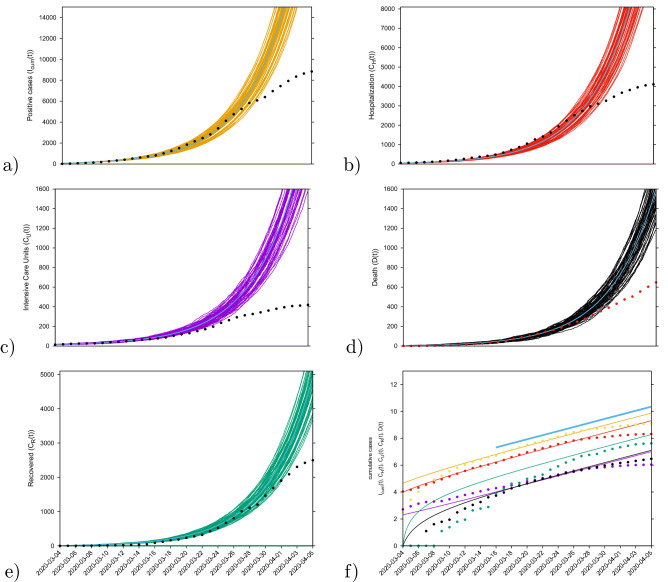
Ensemble of stochastic realizations of the SHARUCD-type model.The mean field solution is shown in light blue. Empirical data are plotted as black/red dots. (**a**) Cumulative tested positive cases $$I_{cum} (t) $$ (yellow lines), (**b**) cumulative hospitalized cases $$C_H (t) $$ (red lines), (**c**) cumulative ICU admission $$C_U (t) $$ (purple lines), (**d**) cumulative deceased cases *D*(*t*) (black lines), and (**e**) cumulative recorded recovered $$C_R(t) $$ (green lines). (**f**) Semi-logarithmic plot of the data and the mean field curves of all variables. For quite some time all mean field curves and the data are in parallel, and we could calculate the slope from the model parameters as growth rate $$\lambda $$, see light blue line.

Parameters are estimated and fixed as the model is able to describe the disease incidence during the exponential phase of the outbreak, see Table 1 in the supplementary material. The stochastic realizations of the model are calculated via the Gillespie algorithm^39,47,48^, which is considered an exact algorithm once the governing equation, the master equation, is specified. Figure 2 shows the ensemble of stochastic realizations of the SHARUCD-type model and data, starting from March 4, 2020 until April 4, 2020. The period in time where the empirical data can no longer be described by the model simulations without control refers to the end of the exponential phase of the epidemic where the exponential growth decelerates into a growth close to zero towards a linear phase.

Our model is able to describe the dynamics observed for each dynamical class, including the observed recovered $C_R$, for which data were only later available. Although the $C_R$ class was not used to determine the parameters, the data on the recorded recovered was immediately described, following the behavior observed from the other classes and without needing to adjust the previously estimated recovery rate $\gamma$.

### Parameter uncertainties and model limitations

**Figure 3:**
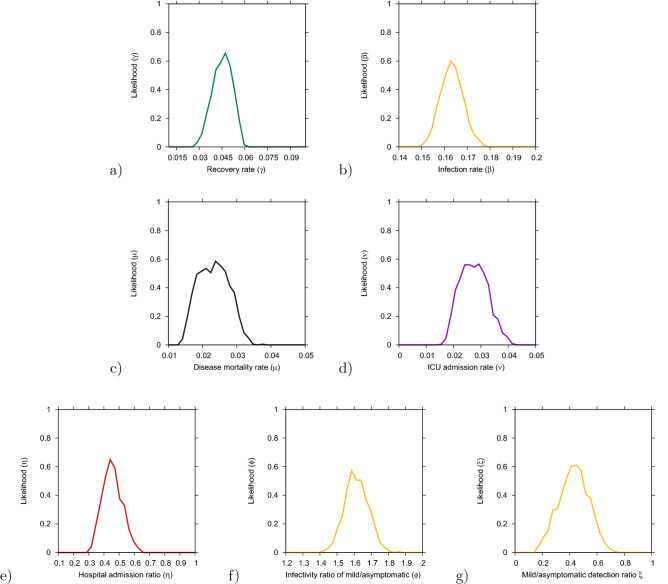
Numerical likelihood functions for the parameters (**a**) recovery rate $\gamma$, (**b**) infection rate $\beta$, (**c**) diseased induced mortality rate $\mu$ and (**d**) rate of ICU facilities admission $\nu$, and (**e**) hospital admission ratio $\eta$, (**f**) infectivity of mild/asymptomatic relative to the hospitalized $\phi$ and (**g**) recording rate of mild/asymptomatic cases $\xi$. Parameter values are presented in Table 1 (supplementary material).

To investigate the parameter insecurities, we calculate numerically likelihood functions^49^ for each parameter conditioned on other parameters and the data, with distances between simulations and data from all 5 variables, *D*(*t*) , $$I_{cum} (t) $$, $$C_H(t)$$, $$C_U(t) $$ and $$C_R (t)$$, evaluated for the exponential phase of the epidemic. The likelihood plots for each individual parameter, recovery rate ($$\gamma $$), infection rate ($$\beta $$), disease induced mortality rate ($$\mu $$), rate of ICU admissions ($$\nu $$) and ratio of hospital admission due disease severity ($$\eta $$), the difference in infectivity of asymptomatic and hospitalized ($$\phi $$) and the detection ratio of mild/asymptomatic infections ($$\xi $$) are shown in Fig. 3. Good agreement of the local maxima of the likelihood functions are obtained. The basic epidemiological parameters are shown in Fig. 3a–d and the more internal ones, concerning the differences between mild and severe disease, are shown in Fig. 3e–g.

Although the parameter set used to describe the exponential phase of COVID-19 outbreak in the Basque Country are coinciding well with the calculated maximum likelihood values (see Table 1 in the supplementary material), parameters are prone to correlations, often only determined as combinations but not individually on scarce data. Possible correlations are investigated using two parameter likelihood plots (see supplementary material). Models output are based on the data available, which are often incomplete, and for non-existing data such as data referring to the proportion of undetected asymptomatic infected as well as how infective those individuals are (i.e. their contribution to the force of infection as compared to the symptomatic detected individuals) during the exponential phase of the epidemic are estimated but not yet validated with empirical data. However, these data would eventually change the dynamical behavior obtained for the positive cases and recovered and for the other variables it would remain the same. As part of the BTMF initiative, we keep calibrating the model framework with real time updated data. Results are updated and publicly available on the “SHARUCD dashboard”^50^, with the selected parameter set continually used also during the control phase without need of adjustments.

### Modelling the effects of the control measures

With the initial parameters estimated and fixed on the exponential phase of the epidemic, we model the effect of the disease control measures introduced using a standard sigmoid function $\sigma (x)=1/(1+e^{x})$, shown along the main results presented in “Momentary growth rates and momentary reproduction ratio” section, which is able to describe well the gradual slowing down of the epidemic, as it turned out later in the response of the disease curves to the control measures, see “Momentary growth rates and momentary reproduction ratio section, and its detailed description below. Specifically, the infection rate $\beta$ becomes time-dependent with $\beta (t)= \beta _0 \sigma _{-}(x(t)) + \beta _1 \sigma _{+} (x(t))$, where $\sigma _{-}:= 1/(1+e^{x})$ is a downward sigmoidal and $\sigma _{+}:= 1/(1+e^{-x})$ is an upward sigmoidal. The time-depend argument is $x(t):=a(t-t_c)$, with $a=0.38 \; d^{-1}$ and $t_c=26$ days after the initial time $t_0$. Modifications of the lower value of infectivity $\beta _1$ might be needed when the relaxation of the control measures can change the contact probabilities again or in the eventual case of seasonality as observed in other respiratory diseases.

The prediction exercises shown in the following sections refers to the original modelling framework presented in “Modelling framework” section.

#### Short-term prediction exercise with control measure

**Figure 4:**
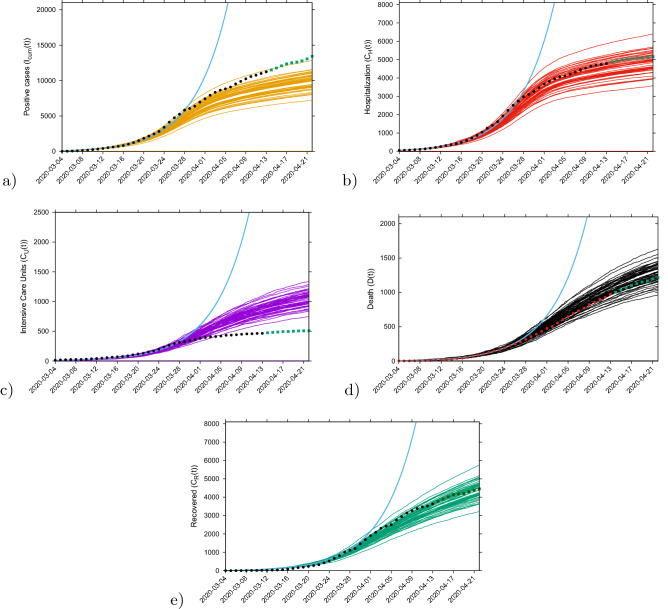
Ensemble of stochastic realizations of the SHARUCD-model with control and data matching, starting from March 4, 2020. The mean field solution without control is shown in light blue. Empirical data from March 4 to April 13, 2020 are plotted as black/red dots. Empirical data from April 14 to April 21, 2020 are plotted a green squares. For short-term predictions. (**a**) Cumulative tested positive cases $$I_{cum} (t) $$ (yellow lines), (**b**) cumulative hospitalized cases $$C_U (t) $$ (red lines), (**c**) cumulative ICU admission $$C_U (t) $$ (purple lines), (**d**) cumulative deceases cases *D*(*t*) (black lines), and (**e**) cumulative recovered $$C_R(t) $$ (green lines).

Short-term predictions considering the effective control measures described above are shown in Fig. 4. For this exercise, empirical data (black/red dots) were available up to April 13, 2020, and model simulations are obtained for seven days longer run than the available data. A week later, new data were included to check the quality of the short prediction exercise (see Fig. 4, green/dark green squares following the black/red dots). The mean field solution without the control function is plotted in light blue, indicating the differences of model prediction with and without control measures. In good agreement, hospitalization and deceased cases are well matched within 50 stochastic simulations obtained with the Gillespie algorithm, with data lying in the median range of stochastic realizations. Note that for the likelihood functions we use several hundreds of stochastic realizations. The ICU admission data look atypical and can not be described with the current control scenario, after the exponential phase of the epidemic. This aspect is investigated in more detail in the following sections, using further measures and model refinements.

The cumulative incidences for tested positive cases ($I_{cum}$) follow the higher realizations range whereas the cumulative incidences for alive hospital discharges ($C_R$), a proxy for notified recovered individuals which were hospitalized because of COVID-19, but not including the recovered individuals which were eventually tested positive but did not need hospitalization, follow the lower realizations range. While the deviation observed for the total tested positive ($I_{cum}$) can be explained by the increased testing capacities over time since March 22, 2020, where more cases are expected to be detected, including sub-clinical infections and eventually asymptomatic individuals, the deviation observed for the “recovered” individuals are as expected, since the dynamical variable $C_R$ counts, besides the notified $H+U$ alive discharges, a proportion of tested positive mild/asymptomatic individuals that did not need hospitalization ($\xi A$).

#### Long-term prediction with control measures

**Figure 5:**
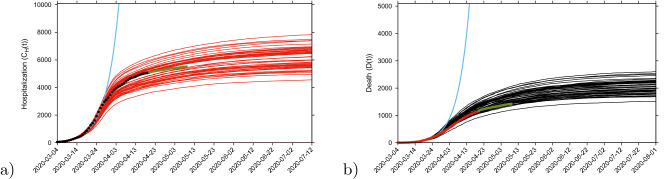
Ensemble of stochastic realizations of the SHARUCD-model. The mean field solution without control is shown in light blue. Empirical data, from March 4 to April 20, 2020 are plotted as black/red dots. Empirical data, from April 21 to May 9, 2020 are plotted as green dots. For long term predictions, (**a**) cumulative hospitalized cases $$C_H (t) $$ (red lines, up to July 12, 2020) and (**b**) cumulative deceases cases *D*(*t*) (black lines, up to August 1, 2020). The scale used here is 1:2 for hospitalizations and deceased cases.

Longer-term predictions for hospital admission and deceased cases are obtained in Fig. 5, with very small numbers of new hospital admission cases (close to zero increment) around 130 days after March 4, 2020. Deceased cases are predicted to reach zero increment 2-3 weeks later, due to the delay between the onset of symptoms, hospitalization and death. Data used in this prediction exercise were available until April 20, 2020 and were not discriminated by diagnostic method (PCR or rapid tests). Therefore, the final numbers might contain some overlap and possible double notification occurring since March 22, when testing capacity gradually stated to increase, including rapid tests, introduced on April 6, 2020, used as screening tool in the Basque Country.

We plot the data continuation with the same criteria as described above, up to May 9, 2020 (green squares), to show that the predictions were well within the $95\%$ confidence intervals of the stochastic realizations. It is important to mention that data collection criteria were updated on May 10, 2020, to include cases with positive PCR only. The parameter set presented here would rather overestimate the number of “confirmed cases by PCR only” as a significant difference is observed in all variables, with a $6\%$ and $3\%$ decrease in hospitalizations and deceased respectively. To describe these updated data set, a model re-parametrization, which is outside the aim of this article, would be needed.

It is important to mention that data collection criteria were updated on May 10, 2020, to include cases with positive PCR only. The current parameter set would rather overestimate the number of “confirmed cases by PCR only”as a significant difference is observed in all variables, with a $6\%$ and $3\%$ decrease in hospitalizations and deceased respectively. To describe these updated data set, a model re-parametrization would be needed.

### Growth rate and reproduction ratio

After an introductory phase, the epidemic entered into an exponential growth phase, which started in the Basque Country around March 10, 2020, and due to the effects of the imposed control measures has left the exponential growth phase to a slower growth around March 27, 2020, see Fig. 2.

The exponential growth phase is typical for any outbreak with disease spreading in a completely susceptible population, as observed already in the SIR-system, Eq. (1), with $\frac{dI}{dt} = \left( \beta \frac{S}{N}-\gamma \right) \cdot I =: \lambda \cdot I$ for $S\approx N$ and hence $\lambda = \frac{d}{dt} \; ln(I)$. For the present SHARUCD model we obtain similarly an exponential growth factor analytically. From the active disease classes *H* and *A* with the dynamics given by

5 $$\begin{aligned} \frac{d}{dt} \left( \begin{array}{c} H\\ A \end{array} \right) = \left[ \left( \begin{array}{cc} \eta \beta \frac{S}{N} &{} \phi \eta \beta \frac{S}{N}\\ (1-\eta ) \beta \frac{S}{N} &{} \phi (1-\eta ) \beta \frac{S}{N} \\ \end{array} \right) - \left( \begin{array}{cc} (\gamma + \mu +\nu ) &{} 0\\ 0 &{} \gamma \\ \end{array} \right) \right] \cdot \left( \begin{array}{c} H\\ A \end{array} \right) \end{aligned}$$

we obtain $\lambda _{1/2} = \frac{1}{2} \cdot tr \pm \sqrt{ \frac{1}{4} \cdot tr^2 - det}$ with the parameter dependent trace $tr = (\eta + \phi (1-\eta )) \cdot \beta - (2\gamma +\mu +\nu )$ and determinant $det = \gamma (\gamma +\mu +\nu )- ((\gamma +\mu +\nu )\phi (1-\eta ) +\gamma \eta ) \cdot \beta$. The dominating growth factor is then given by the largest eigenvalue $\lambda _1$. The concept of the growth rate can be extended into the phase when effects of the control measures become visible and parameters slowly change, such that for short times the above analysis holds as for constant parameters.

Another measure of the spreading of the disease in its initial phase is the basic reproduction number ($$R_0$$), the number of secondary cases $$I_s $$ from a primary case $$I_p $$ during its infectivness before recovering in a completely susceptible population, giving in the SIR dynamics reproduction ratio $$r=\beta /\gamma $$.

From the next generation matrix in the case of the SHARUCD model we obtain the dominant eigenvalue $r_1 = \frac{\eta \gamma + (1-\eta ) \phi (\gamma + \mu +\nu )}{\gamma (\gamma + \mu +\nu )} \cdot \beta$ as the reproduction ratio in a completely susceptible population. This concept can be also extended to larger compartmental models and into the phase when effects of the control measures become visible and parameters slowly change. The momentary reproduction ratios *r* can be analyzed, as frequently done for the COVID-19 epidemics, but often called “R”. While the momentary growth rate follows directly from the time continuous data at hand, the momentary reproduction ratio depends on the notion of a generation time $\gamma ^{-1}$. The momentary growth rates and momentary reproduction ratio are analyzed below.

#### Momentary growth rates and momentary reproduction ratio

We calculate the growth rates and reproduction numbers for the Basque Country from the COVID-19 data at hand available, from March 4 to May 4, 2020. As the absolute value of *r*(*t*) is bound to many internal assumptions, recovery period, smoothing and approximations, which are not valid for time-dependent parameters, the threshold behavior is independent of those uncertainties and clearly indicates that the outbreak gets under control from April onwards, allowing a gradual lockdown lifting restrictions under constant monitoring. Note that this analysis has terminated on May 4, 2020, before the lockdown started to be lifted in the Basque Country.

**Figure 6:**
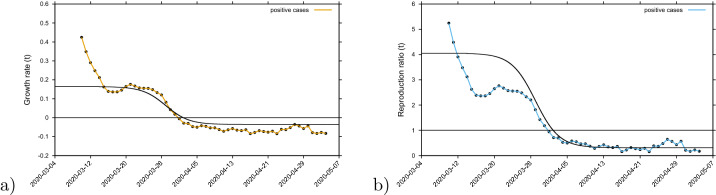
(**a**) Growth rate estimation from the data on positive tested infected cases, and (**b**) reproduction ratio from the same data. With $\Delta t=5$ days and $\tau =7$ days, the sigmoidal black curve represents the values of $\lambda _1$ and $r_1$ computed on the basis of $\beta (t)$.

To obtain the momentary growth rates from data directly we use $$\lambda = \frac{d}{dt} \; ln(I)$$ at first applied to the cumulative tested positive cases $$I_{cum} (t) $$ obtaining, via a smoothing window, the new cases after time $$\tau $$ as

6 $$\begin{aligned} I_{new,\tau } (t) := I_{cum} (t) - I_{cum} (t-\tau ) \end{aligned}$$

and hence, the growth rate

7 $$\begin{aligned} \lambda = \frac{1}{\Delta t} \left( \frac{}{} ln(I_{new,\tau } (t)) - ln(I_{new,\tau } (t-\Delta t)) \right) \quad . \end{aligned}$$

From the growth rate, the reproduction ratio is calculated with the recovery period $$\gamma ^{-1}$$^51^ obtained from underlying models and recent literature about SARS-CoV-2 interaction with human hosts^26,27,43–46^. The results are given as data dots in Fig. 6a for the growth rates and in Fig. 6b for the reproduction ratios and the black curves from the control response $$\beta (t) $$ with its sigmoidal shape (black line) using the SHARUCD-model expressions $$\lambda _1 $$ in Fig. 6a and $$r_1 $$ in Fig. 6b. After an original introductory phase of the epidemic where insecurities in the data collection (due to small numbers) were still present while setting up the recording system, the curves agree well, from around March 14, 2020 on, with surprisingly good results, also in the lower value of the sigmoidal curve.

**Figure 7:**
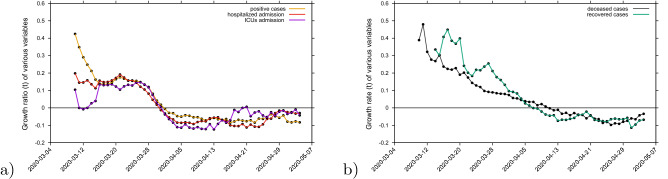
Growth rate estimation for various variables. (**a**) Growth rate for tested positive cases (yellow), hospitalizations (red) and ICU (purple). (**b**) Growth rate for recovered (green) and deceased (black). Two groups of growth behaviour in response to the lockdown measures are observed,’crossing the threshold of zero growth at different times.

The concept of growth rates can be extended to the other measured variables, hospitalizations, deceased cases, recovered cases and ICU admitted cases, see Fig. 7. The sigmoidal shape of decreasing growth rates is well visible in the hospitalized and the tested positive infected, whereas the deceased and the recovered are following only later, and a much slower sigmoidal curve or near to linear decline. Notably, the ICU admitted cases follow the sharp sigmoidal decline of growth rate of hospitalized and tested positive cases rather than the growth rate of deceased and the recovered.

Analysis of the momentary reproduction ratio and momentary growth rates^51^ have shown two groups of growth behaviour in response to the lockdown measures. Synchronization of the ICU admission cases with the cumulative tested positive cases and hospitalizations was observed, following the sigmoidal function behaviour, and the deceased and recovered cases showing a delay in response to the control measures of 8 to 10 days. These results led to information about how to refine the model in order to capture the dynamics of the ICU admissions better than in the present model as we will describe in the next subsection.

### Model refinement based on observations from growth rates of the different data sets

Given the observed synchronization of the ICU admission cases with the cumulative tested positive cases and hospitalizations, the SHARUCD model is now refined, using data available from March 4 to May 4, 2020.

While in the original model “severity of disease was decided” upon infection with a proportion of infected individuals $\eta$ becoming severely ill prone to hospitalization, the transition into ICU admissions is now changed to a ratio, with infection causing from asymptomatic up to very severe cases, with the assumption that ICU admissions are results of infection like as asymptomatic or hospitalization and not progression from hospitalization.

**Figure 8:**
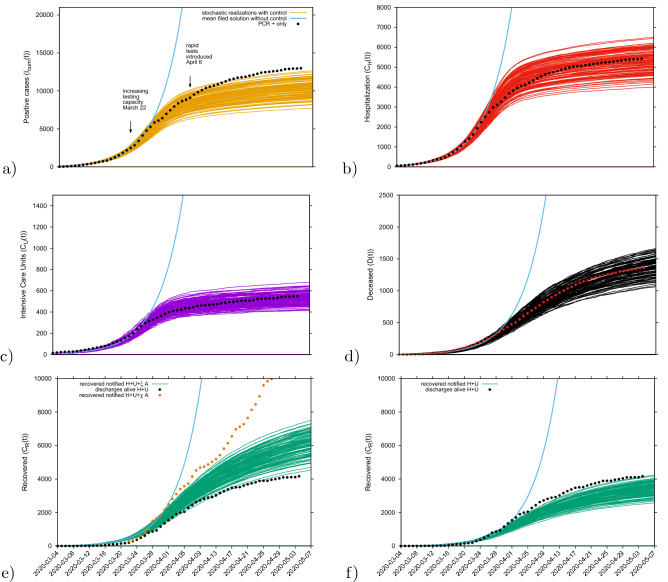
Ensemble of stochastic realizations of the refined SHARUCD-model and data matching. The mean field solution without control is shown in light blue. Empirical data are plotted as black/red dots. (**a**) Cumulative tested positive cases $$I_{cum} (t) $$, (**b**) cumulative hospitalized cases $$C_U (t) $$, (**c**) cumulative ICU admissions $$C_U (t) $$, (**d**) cumulative deceases cases *D*(*t*) , (**e**, **f**) cumulative recovered $$C_R(t) $$.

The updated transitions are changed from the previously used form

8 $$\begin{aligned} \begin{array}{ll} w_1 ({\underline{x}}) = \eta \beta x_1 (x_2+\phi x_3+\varrho ) , &{} {\underline{r}}_1 = (1,-1,0,0,0,-1,0,0,0,0)^{tr} \\ w_7 ({\underline{x}}) = \nu x_2 , &{} {\underline{r}}_7 = (0,1,0,0,-1,0,0,-1,0,0)^{tr} \end{array} \end{aligned}$$

into

9 $$\begin{aligned} \begin{array}{ll} w_1 ({\underline{x}}) = \eta (1-\nu ) \beta x_1 (x_2+\phi x_3+\varrho ) , &{} {\underline{r}}_1 = (1,-1,0,0,0,-1,0,0,0,0)^{tr} \\ w_7 ({\underline{x}}) = \eta \nu \beta x_1 (x_2+\phi x_3+\varrho ) , &{} {\underline{r}}_7 = (1,0,0,0,-1,-1,0,-1,0,0)^{tr} \end{array} \end{aligned}$$

with the parameter $$\nu $$ adjusted from the ICU-admission rate in units of $$d^{-1} $$ into an ICU-admission ratio $$\nu \in [0,1]$$. By changing and fixing $$\nu =0.1 $$, we obtained immediately a very good agreement between the available cumulative ICU data and model simulations, with only small deviations in the other variables, see Fig. 8c.

In good agreement, the refined model can describe well the hospitalizations (see Fig. 8b), the ICU admissions and the deceased cases (see Fig. 8d), well matched within the median of the 100 stochastic realizations from the model. The cumulative incidences for tested positive cases (RT-PCR+ only) still follow the higher realizations range (see Fig. 8a), due to the increasing testing capacities since March 22, 2020, followed by the introduction of rapid tests, on April 6, 2020, as screening tool that the current model (without testing feedback) can not describe quantitatively. The cumulative incidences for alive hospital discharges (black dots), used as a proxy for notified recovered individuals needing hospitalization because of COVID-19 only, keeps following the lower realizations range, when model simulations are obtained for the cumulative notified recovered ($H+U+\xi A$), shown in Fig. 8e. Orange dots refers to all notified recovered ($H+U+\chi A$), including also tested positive mild/asymptomatic infections detected when the testing capacity increased. To describe this new data set, a new transition is needed to record the proportion of recovered from detected mild/asymptomatic infections ($\chi$). Figure 8f shows model simulations obtained for the cumulative notified recovered from hospital ($H+U$) only. Similarly, in order to describe quantitatively this specific available data set, a new transition is needed to record a proportion of recovered individuals tested positive without being admitted to the hospital ($\xi A$).

**Figure 9:**
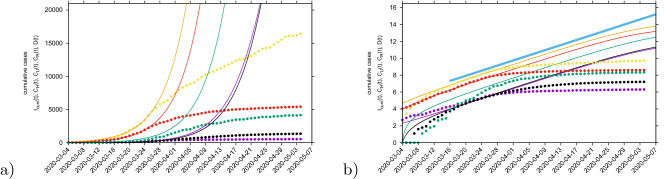
Adjusted SHARUCD model to synchronize ICU admissions with tested positive cases and hospitalizations. (**a**) Data and mean field solutions in natural scale. (**b**) Data and mean field solutions in semi-log scale with adjusted mean field curves and growth rate $\lambda$ as light blue line.

The calculations of the growth factors and reproduction ratios were updated to the new model modifications, namely from the disease class dynamics now as

10 $$\begin{aligned} \frac{d}{dt} \left( \begin{array}{c} H\\ A \end{array} \right) = \left[ \left( \begin{array}{cc} \eta (1-\nu ) \beta \frac{S}{N} &{} \phi \eta (1-\nu ) \beta \frac{S}{N}\\ (1-\eta ) \beta \frac{S}{N} &{} \phi (1-\eta ) \beta \frac{S}{N} \\ \end{array} \right) - \left( \begin{array}{cc} (\gamma + \mu ) &{} 0\\ 0 &{} \gamma \\ \end{array} \right) \right] \cdot \left( \begin{array}{c} H\\ A \end{array} \right) \end{aligned}$$

we obtain $\lambda _1$ via $\lambda _{1/2} = \frac{1}{2} \cdot tr \pm \sqrt{ \frac{1}{4} \cdot tr^2 - det}$ with $tr = (\eta (1-\nu )+ \phi (1-\eta )) \cdot \beta - (2\gamma +\mu )$ and $det = \gamma (\gamma +\mu )- ((\gamma +\mu )\phi (1-\eta ) +\gamma \eta (1-\nu )) \cdot \beta$ and the reproduction ratio as $r_1 = \frac{\eta (1-\nu ) \gamma + (1-\eta ) \phi (\gamma + \mu )}{\gamma (\gamma + \mu )} \cdot \beta$. From Fig. 9 we observe the data to follow the exponential growth phase of COVID-19 epidemic in the Basque Country from about March 14 to March 27, 2020, sensing the slow down due to the control measures from March 28, 2020 onwards. Figure 9b shows the semi-logarithmic plot for the refined model and its adjusted parameter set matching with the data for all variables. The analytically calculated growth rate $\lambda _1$ (light blue line) is also shown, close to the mean field solutions (parallel straight lines) for the exponential phase of the outbreak.

#### Prediction up to May 25th

**Figure 10:**
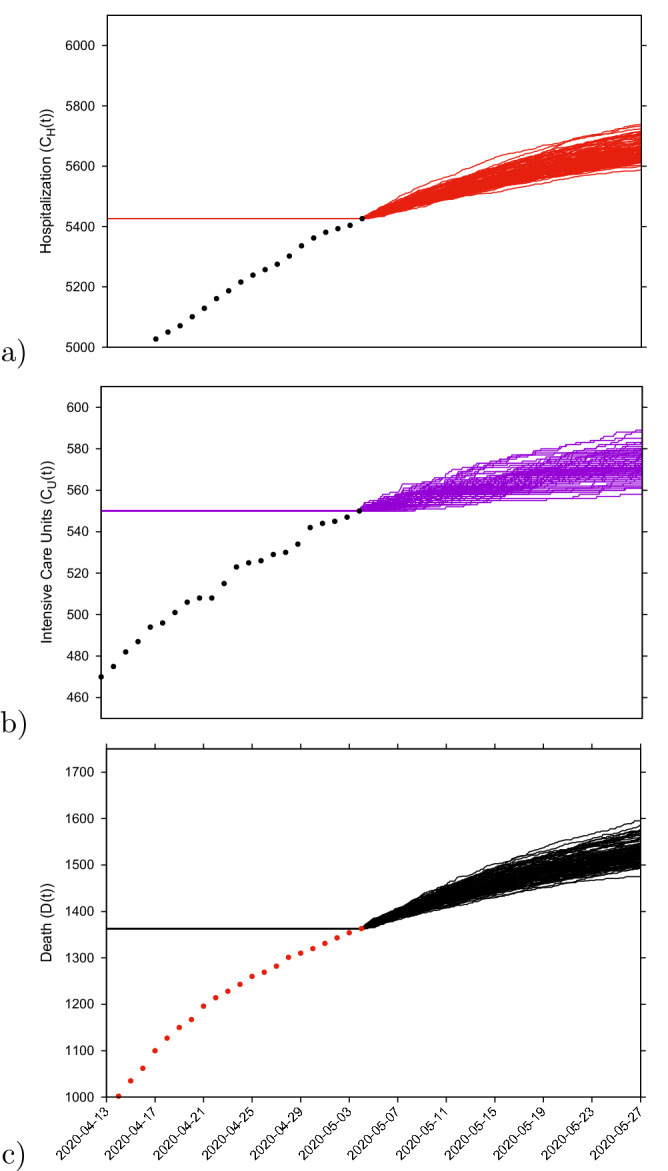
(**a**) Cumulative hospitalized cases $$C_H(t) $$ (red lines), (**b**) cumulative ICU admissions $$C_U(t)$$ (purple lines), (**c**) cumulative deceased cases *D*(*t*) (black lines). Empirical data are plotted, up to May 4, 2020, as black/red dots.

Medium-term prediction exercise under constant external conditions was performed using the last data point available on May 4, 2020. Taking the minimum and the maximum ranges of the stochastic realizations as reference, the number of new hospitalizations, shown in Fig. 10a, is predicted to be between $\approx$ 5600 to $\approx$ 5750 cumulative cases up to May 25, 2020 and ICU admissions, shown in Fig. 10b, between $\approx$ 560 to $\approx$ 590 cumulative cases up to May 25, 2020. The number of deceased cases, shown in Fig. 10c, is predicted to be between $\approx$ 1500 to $\approx$ 1600 by May 25th, referring to the number of predicted confirmed severe cases from 2-3 weeks before (May 5th), due to the delay between the onset of symptoms, hospitalization and death. Note that the data used for these predictions did not differentiate the method used for disease cases notification, with possible double notification occurring when the rapid tests were introduced as screening tool.

As described in “Long-term prediction with control measures” section, we recall that data collection criteria were updated on May 10, 2020 (after this manuscript was submitted), to include cases with positive PCR only. The current parameter set would rather overestimate the number of “confirmed cases by PCR only”as a significant difference is observed in all variables, with a $6\%$ and $3\%$ decrease in hospitalizations and deceased respectively. To describe these updated data set, a model re-parametrization would be needed.

## Discussion and future work

In March 2020, a multidisciplinary task force (so-called Basque Modelling Task Force, BMTF) was created to assist the Basque Health managers and the Basque Government during the COVID-19 responses. In this manuscript, we presented the BMTF efforts while developing a flexible modeling framework, allowing refinements as new data and knowledge becomes available. As a follow up exercise, we describe the steps considered from March 4 up to May 4, while the epidemic was unfolding and the control measures were implemented.

We have analyzed the results obtained with a stochastic SHARUCD-type model framework, taking into consideration all information provided by the public health frontline and keeping the biological parameters for COVID-19 in the range of the recent research findings, but adjusting to the phenomenological data description. Using the data provided by the Basque Health Service, models were able to describe the disease incidence data with a single parameter set.

A careful data inspection has shown the end of the exponential phase of the epidemic to be around March 26, 2020, allowing us to infer that the partial lockdown was effective and enough to decrease disease transmission in the Basque Country. Disease control was modeled, given the current epidemiological scenario, able to describe well the gradual slowing down of the COVID-19 outbreak. Short-term prediction exercises were performed, with seven days longer simulation runs than the available data, where hospitalizations, recovered and deceased cases matched well within the median of the stochastic model simulations. The ICU admission data looked atypical under the baseline proposed model and could only be described qualitatively during the exponential phase of the outbreak, however not well quantified afterwards. The cumulative incidence for all positive cases followed the higher stochastic realizations range instead and the deviation observed between model simulations and data was interpreted by the increased testing capacities with not only the expected increment in the number of new positive cases, including sub-clinical infections and eventually asymptomatic individuals, but also with some overlap and possible double notification occurring when the rapid tests were introduced as screening tool in nursing homes and for the frontline public health workers.

Growth rates ($\lambda (t)$) and the reproduction ratio (*r*) were calculated from the model and from the data and the momentary reproduction ratio *r* was estimated to be below the threshold behavior of $r=1$. Although, by May 4, 2020, the number of new cases reported in the Basque Country were decelerating, the outbreak was still in its linear growth phase and careful monitoring of the development of the dynamics of the new cases from all variables and respectively all data sets is required. The growth rates for various variables are negative, confirming the momentary decrease in disease transmission. Moreover, we observed that the dynamics of the hospitalizations and ICU admissions were synchronized with the total tested positive cases, becoming negative on April 1, 2020, whereas recovered and deceased cases only follow later, reaching negative growth rate on April 7 and April 11, 2020, respectively. These results led to a model refinement to synchronize ICU admissions to hospitalizations and to positive tested infected, rather than to deceased and to recovered. The refined model is now able to describe well the 5 variables, with the empirical data lying in the median range of stochastic realizations using a single parameter set.

The value of the momentary reproduction number is affected by a large number of different factors, many of which are difficult to accurately estimate from data. Therefore, particular caution should be used when interpreting momentary reproduction numbers alone for the purposes of policy decisions. The BMTF is monitoring the development of the COVID-19 epidemic in the Basque Country by also evaluating the momentary growth rates $\lambda (t)$ for the positive cases $I_{cum}(t)$, but also the $\lambda (t)$ for hospitalizations ($C_H$), ICU admissions ($C_U$), deceased (*D*) and recovered cases ($C_R$). Without interfering in any political decision, we assist the Basque Health Managers and the Basque Government with results that are obtained by this model framework, based on available data and evidence as scientific advice.

A mid-term prediction exercise using the refined framework was shown , under constant external conditions, with a note of wide confidence intervals considering the higher ranges of model realizations. Models limitations and the implication of using different available data sets were discussed.

As future work, a slight adjustment of the model could further improve the description of the tested positive cases dynamics and the recovered via testing feedback. This will become more important in the future course of the epidemic and will give us better information on the level of asymptomatic and mild infections, allowing to infer on population immunity development in the course of the year and eventually the following years.

## Supplementary information

Supplementary Information.

## References

[1] World Health Organization. *Naming the Coronavirus Disease (COVID-19) and the Virus that Causes it*. Retrieved from https://www.who.int/emergencies/diseases/novel-coronavirus-2019/technical-guidance/naming-the-coronavirus-disease-(covid-2019)-and-the-virus-that-causes-it.

[2] World Health Organization. *Emergencies Preparedness, Response*. Novel Coronavirus China. Retrieved from https://www.who.int/csr/don/12-january-2020-novel-coronavirus-china/en/.

[3] World Health Organization. *WHO Announces COVID-19 Outbreak a Pandemic*. Retrieved from http://www.euro.who.int/en/health-topics/health-emergencies/coronavirus-covid-19/news/news/2020/3/who-announces-covid-19-outbreak-a-pandemic.

[4] World Health Organization. *Coronavirus Disease (COVID-2019) Situation Reports*. https://www.who.int/docs/default-source/coronaviruse/situation-reports/20200420-sitrep-91-covid-19.pdf?sfvrsn=fcf0670b_4.

[5] Governo Italiano Presidenza del Consiglio dei Ministri, March 9th, 2020. Retrieved from http://www.governo.it/it/articolo/firmato-il-dpcm-9-marzo-2020/14276.

[6] Ministerio de la Presidencia, Relaciones con las Cortes y Memoria Democrtica, March 14th, 2020. Retrieved from http://noticias.juridicas.com/base_datos/Laboral/661797-rd-463-2020-de-14-mar-estado-de-alarma-para-la-gestion-de-la-situacion-de.html.

[7] Ministerio de la Presidencia, Relaciones con las Cortes y Memoria Democrtica, March 27, 2020. Retrieved from http://noticias.juridicas.com/base_datos/Admin/662751-real-decreto-476-2020-de-27-de-marzo-por-el-que-se-prorroga-el-estado-de.html.

[8] Ministerio de la Presidencia, Relaciones con las Cortes y Memoria Democrtica, March 29, 2020. Retrieved from http://noticias.juridicas.com/base_datos/Laboral/662759-rd-ley-10-2020-de-29-mar-regulacion-de-un-permiso-retribuido-recuperable.html.

[9] Boletín oficial: BOPV (Pas Vasco). DECRETO 6/2020, de 13 de marzo, del Lehendakari. Retrieved from https://www.legegunea.euskadi.eus//eli/es-pv/d/2020/03/13/6/dof/spa/html/x59-contfich/es/.

[10] Ministerio de la Presidencia, Relaciones con las Cortes y Memoria Democrtica, April 10th, 2020. Retrieved from https://www.who.int/emergencies/diseases/novel-coronavirus-2019/technical-guidance/naming-the-coronavirus-disease-(covid-2019)-and-the-virus-that-causes-it0.

[11] Ministerio de la Presidencia, Relaciones con las Cortes y Memoria Democrática, April 10th, 2020. Retrieved from https://www.who.int/emergencies/diseases/novel-coronavirus-2019/technical-guidance/naming-the-coronavirus-disease-(covid-2019)-and-the-virus-that-causes-it1.

[12] de España, Gobierno. Plan para la Transicin hacia una nueva normalidad, April 28th. Retrieved from https://www.who.int/emergencies/diseases/novel-coronavirus-2019/technical-guidance/naming-the-coronavirus-disease-(covid-2019)-and-the-virus-that-causes-it2 (2020).

[13] Anderson Roy M, Heesterbeek Hans, Don Klinkenberg T, Hollingsworth Déirdre (2020). How will country-based mitigation measures influence the course of the COVID-19 epidemic?. The Lancet.

[14] Imperial College COVID-19 Response Team. Estimating the number of infections and the impact of non-pharmaceutical interventions on COVID-19 in 11 European countries. March 30th, 2020. Retrieved from https://www.who.int/emergencies/diseases/novel-coronavirus-2019/technical-guidance/naming-the-coronavirus-disease-(covid-2019)-and-the-virus-that-causes-it3.

[15] Kissler Stephen M, Tedijanto Christine, Goldstein Edward, Grad Yonatan H, Lipsitch Marc (2020). Projecting the transmission dynamics of SARS-CoV-2 through the postpandemic period. Science.

[16] Aguiar M, Paul R, Sakuntabhai A, Stollenwerk N (2014). Are we modeling the correct data set? Minimizing false predictions for dengue fever in Thailand. Epidemiol. Infect..

[17] Anastassopoulou C, Russo L, Tsakris A, Siettos C (2020). Data-based analysis, modelling and forecasting of the COVID-19 outbreak. PLoS ONE.

[18] Michaud, J., Kates, J., & Levitt, L. *COVID-19 Models: Can They Tell Us What We Want to Know?* Retrieved from Apr 16 2020 https://www.kff.org/coronavirus-policy-watch/covid-19-models/.

[19] Giordano Giulia (2020). Modelling the COVID-19 epidemic and implementation of population-wide interventions in Italy. Nat. Med..

[20] Hauser A (2020). Estimation of SARS-CoV-2 mortality during the early stages of an epidemic: a modeling study in Hubei, China, and six regions in Europe. PLoS Med..

[21] Kyrychko, Y., Blyuss, K., & Brovchenko, I. Mathematical modelling of dynamics and containment of COVID-19 in Ukraine. Preprint at 10.1101/2020.07.24.20161497. 10.1038/s41598-020-76710-1 PMC7665000 33184338

[22] Yi Y, Lagniton PN, Ye S, Li E, Xu RH (2020). COVID-19: what has been learned and to be learned about the novel coronavirus disease. Int. J. Biol. Sci..

[23] Kramer, M. *et al.**Epidemiological Data from the nCoV-2019 Outbreak: Early Descriptions from Publicly Available Data*. Retrieved from http://virological.org/t/epidemiological-data-from-the-ncov-2019-outbreak-early-descriptions-from-publicly-available-data/337.

[24] Wu P (2020). Real-time tentative assessment of the epidemiological characteristics of novel coronavirus infections in Wuhan, China, as at 22 January 2020. Euro Surveill..

[25] Guan Wei-jie (2020). Clinical characteristics of coronavirus disease 2019 in China. N. Engl. Med. J..

[26] Lauer Stephen A (2020). The incubation period of coronavirus disease 2019 (COVID-19) from publicly reported confirmed cases: estimation and application. Ann. Intern. Med..

[27] Liu Yang (2020). Viral dynamics in mild and severe cases of COVID-19. Lancet. Infect. Dis.

[28] Aguiar M, Kooi WB, Rocha F, Ghaffari P, Stollenwerk N (2013). How much complexity is needed to describe the fluctuations observed in dengue hemorrhagic fever incidence data?. Ecol. Complex..

[29] Aguiar M, Ballesteros S, Kooi BW, Stollenwerk N (2011). The role of seasonality and import in a minimalistic multi-strain dengue model capturing differences between primary and secondary infections: complex dynamics and its implications for data analysis. J. Theor. Biol..

[30] Stollenwerk N (2010). A spatially stochastic epidemic model with partial immunization shows in mean field approximation the reinfection threshold. J. Biol. Dyn..

[31] de España, G. Ministerio de Sanidad. Actualización enfermedad por el coronavirus (COVID-19). https://www.mscbs.gob.es/profesionales/saludPublica/ccayes/alertasActual/nCov-China/.

[32] Vasco, G. Departamento de Salud. Informes con la actualización de datos sobre la evolución del nuevo coronavirus COVID-19. https://www.euskadi.eus/boletin-de-datos-sobre-la-evolucion-del-coronavirus/web01-a2korona/es/.

[33] Aguiar M, Stollenwerk N (2020). Condition-specific mortality risk can explain differences in COVID-19 case fatality ratios around the globe. J. Public Health..

[34] Aguiar M, Stollenwerk N (2020). SHAR and effective SIR models: from dengue fever toy models to a COVID-19 fully parametrized SHARUCD framework. Commun. Biomath. Sci..

[35] Duong V (2015). Asymptomatic humans transmit dengue virus to mosquitoes. Proc. Natl. Acad. Sci..

[36] van Kampen NG (1992). Stochastic Processes in Physics and Chemistry.

[37] Gardiner CW (1985). Handbook of Stochastic Methods.

[38] Honerkamp J (1993). Stochastic Dynamical Systems: Concepts, Numerical Methods and Data Analysis.

[39] Stollenwerk N, Jansen V (2011). Population Biology and Criticality: From Critical Birth–Death Processes to Self-Organized Criticality in Mutation Pathogen Systems.

[40] Stollenwerk N (2017). Hopf and torus bifurcations, torus destruction and chaos in population biology. Ecol. Complex..

[41] Gang Hu (1987). Stationary solution of master equations in the large-system-size limit. Phys. Rev. A.

[42] Billings L, Mier-y-Teran-Romero L, Lindley B, Schwartz IB (2013). Intervention-based stochastic disease eradication. PLoS ONE.

[43] Tetro JA (2020). Is COVID-19 receiving ADE from other coronaviruses?. Microbes Infect..

[44] Fu Y, Cheng Y, Wu Y (2020). Understanding SARS-CoV-2-mediated inflammatory responses: from mechanisms to potential therapeutic tools. Virol. Sin..

[45] Lescure Francois-Xavier (2020). Clinical and virological data of the first cases of COVID-19 in Europe: a case series. Lancet Infect. Dis.

[46] Li Ruiyun (2020). Substantial undocumented infection facilitates the rapid dissemination of novel coronavirus (SARS-CoV-2). Science.

[47] Gillespie DT (1976). A general method for numerically simulating the stochastic time evolution of coupled chemical reactions. J. Comput. Phys..

[48] Gillespie DT (1978). Monte Carlo simulation of random walks with residence time dependent transition probability rates. J. Comput. Phys..

[49] Stollenwerk N, Briggs KM (2000). Master equation solution of a plant disease model. Phys. Lett. A.

[50] Epidemiological SHARUCD model. https://wp.bcamath.org/news/en/epidemiological-sharucd-model/.

[51] Aguiar M, Van-Dierdonck JB, Stollenwerk N (2020). Reproduction ratio and growth rates: measures for an unfolding pandemic. PLoS ONE.

