# Supplementary material for: Modelling COVID 19 in the Basque Country from introduction to control measure response
Source: Sci Rep. 2020 Oct 14;10:17306. doi: 10.1038/s41598-020-74386-1 (PMC7560887; doi:10.1038/s41598-020-74386-1)
Supplement: Supplementary file 1 — Supplementary Information. [file 41598_2020_74386_MOESM1_ESM.pdf]

Modelling COVID 19 in the Basque Country

from introduction to control measure response

Maíra Aguiar[1,2,3*], Eduardo Millán Ortuondo[4],
Joseba Bidaurrazaga Van-Dierdonck[5], Javier Mar[6,7,8], and
Nico Stollenwerk[1,9]

[1] *Dipartimento di Matematica, Università degli Studi di Trento, Via Sommarive, 14 38123 Povo (Trento), Italy*
[2] *Basque Center for Applied Mathematics (BCAM), Alameda Mazarredo, 14, 48009 Bilbao, Spain*
[3] *Ikerbasque, Basque Foundation for Science, Bilbao, Spain*
[4] *Osakidetza Basque Health Service, General Sub-directorate for healthcare, Vitoria-Gasteiz, Spain*
[5] *Public Health, Basque Health Department, Rekalde Zumarkalea 39A 48008, Bilbao, Spain*
[6] *Osakidetza Basque Health Service, Debagoiena Integrated Healthcare Organisation, Research Unit, Arrasate-Mondragón, Guipúzcoa, Spain*
[7] *Biodonostia Health Research Institute, Donostia-San Sebastián, Guipúzcoa, Spain*
[8] *Kronikgune Institute for Health Services Research, Economic Evaluation Unit, Barakaldo, Spain.*
[9] *Center for Mathematics, Fundamental Applications and, Operations Research, Lisbon University, Portugal*
[*] Correspondence to m.aguiar@unitn.it

Supplementary Material:

1 Mean field approximation and stochastic differential equation approximation of the basic SHARUCD model

From the master equation for state discrete stochastic processes, given in densities, we derive now a stochastic differential equation system as diffusion approximation, which as by-product also gives the mean field approximation as deterministic drift term in the Fokker-Planck equa-

tion. We use Taylor's expansion for small changes of densities $\Delta \underline{x}_j$, hence

$$w_j(\underline{x} + \Delta \underline{x}_j) \cdot p(\underline{x} + \Delta \underline{x}_j, t) = \sum_{\nu=0}^{\infty} \frac{1}{\nu!} \Big(\Delta \underline{x}_j \cdot \nabla_{\underline{x}} \Big)^{\nu} w_j(\underline{x})\, p(\underline{x}, t) \tag{1}$$

giving to second order in $1/N$ a Fokker-Planck equation

$$\begin{aligned} \frac{\partial}{\partial t}\, p(\underline{x}, t) &= -\nabla_{\underline{x}} \left(\sum_{j=1}^{n} (-\underline{r}_j \cdot w_j(\underline{x}))\, p(\underline{x}, t) \right) \\ &\quad + \frac{\sigma^2}{2} \sum_{j=1}^{n} (\underline{r}_j \cdot \nabla_{\underline{x}})^2 w_j(\underline{x})\, p(\underline{x}, t) \end{aligned} \tag{2}$$

with $\nabla_{\underline{x}} = \left(\frac{\partial}{\partial x_1}, ..., \frac{\partial}{\partial x_{10}} \right)^{tr} = \partial_{\underline{x}}$ or in different notation

$$\frac{\partial}{\partial t}\, p(\underline{x}, t) = -\partial_{\underline{x}} \Big(\underline{f}(\underline{x})\, p(\underline{x}, t) \Big) + \frac{\sigma^2}{2} \overrightarrow{\partial_{\underline{x}}} \Big(G^2(\underline{x})\, p(\underline{x}, t) \Big) \overleftarrow{\partial_{\underline{x}}} \tag{3}$$

using simply a quadratic form $\overrightarrow{\partial_{\underline{x}}}\, (G^2(\underline{x})\, p(\underline{x}, t))\, \overleftarrow{\partial_{\underline{x}}}$ here with

$$\overrightarrow{\partial_{\underline{x}}}\, (G^2\, p)\, \overleftarrow{\partial_{\underline{x}}} = \left(\frac{\partial}{\partial x_1}, ..., \frac{\partial}{\partial x_{10}} \right) \cdot \begin{pmatrix} g_{1,1} & \cdots & g_{1,10} \\ \cdots & \cdots & \cdots \\ g_{10,1} & \cdots & g_{10,10} \end{pmatrix}^2 p(\underline{x}, t) \cdot \begin{pmatrix} \overleftarrow{\frac{\partial}{\partial x_1}} \\ \cdots \\ \overleftarrow{\frac{\partial}{\partial x_{10}}} \end{pmatrix} \tag{4}$$

and

$$\begin{aligned} \underline{f}(\underline{x}) &= \sum_{j=1}^{n} \underline{f}_j(\underline{x}) = \sum_{j=1}^{n} (-\underline{r}_j \cdot w_j(\underline{x})) \\ G^2(\underline{x}) &= \sum_{j=1}^{n} G_j^2(\underline{x}) = \sum_{j=1}^{n} \underline{r}_j \cdot \underline{r}_j^{tr}\, w_j(\underline{x}) \quad . \end{aligned} \tag{5}$$

The Fokker-Planck equation gives a stochastic differential equation system with $\sigma = 1/\sqrt{N}$ and Gaussian normal noise vector $\underline{\varepsilon}(t) = (\varepsilon_{x_1}(t), ..., \varepsilon_{x_{10}}(t))^{tr}$ as

$$\frac{d}{dt}\underline{x} = \underline{f}(\underline{x}) + \sigma G(\underline{x}) \cdot \underline{\varepsilon}(t) \tag{6}$$

and using matrix square root from eigenvalue-eigenvector decomposition $G^2(\underline{x}) = T \Lambda T^{-1}$ as $G(\underline{x}) = T \sqrt{\Lambda} T^{tr}$ to be numerically implemented easily, and much faster than the Gillespie algorithm for the master equation, when it comes to large population sizes N and longer runs

of e.g. one year, as for a complete disease outbreak curve necessary. For possibly non-quadratic matrices B, expressing the covariance matrix as $B^{tr}B = G^2$, see [1], speeding up further the stochastic process simulations.

In mean field approximation we obtain explicitly from

$$\frac{d}{dt}\underline{x} = \underline{f}(\underline{x}) = \sum_{j=1}^{n}(-\underline{r}_j \cdot w_j(\underline{x})) \tag{7}$$

with the transitions $w_j(\underline{x}))$ specified in Eq. 4 in the main text. The deterministic version of the model is given by a differential equation system for all classes, including the recording classes of cumulative cases C_H, C_A, C_R and C_U by

$$\begin{aligned}
\frac{d}{dt}S &= -\beta\frac{S}{N}(H + \phi A + \varrho N) \\
\frac{d}{dt}H &= \eta\beta\frac{S}{N}(H + \phi A + \varrho N) - (\gamma + \mu + \nu)H \\
\frac{d}{dt}A &= (1-\eta)\beta\frac{S}{N}(H + \phi A + \varrho N) - \gamma A \\
\frac{d}{dt}R &= \gamma(H + U + A) \\
\frac{d}{dt}U &= \nu H - (\gamma + \mu)U \\
\frac{d}{dt}C_H &= \eta\beta\frac{S}{N}(H + \phi A + \varrho N) \\
\frac{d}{dt}C_A &= \xi \cdot (1-\eta)\beta\frac{S}{N}(H + \phi A + \varrho N) \\
\frac{d}{dt}C_R &= \gamma(H + U + \xi A) \\
\frac{d}{dt}C_U &= \nu H \\
\frac{d}{dt}D &= \mu(H + U)
\end{aligned} \tag{8}$$

in a complete form.

For a constant population size N, susceptible individuals (S) become infected with SARS-CoV-2. With proportion η, individuals become severe cases requiring hospitalization (H), always detected via positive RT-PCR test or double testing with a first rapid test for antibodies, though up to now being quite unspecific, and a subsequent more specific RT-PCR test, whereas with proportion $1-\eta$ individuals develop mild/asymptomatic infection, which are detected with a ratio ξ ($\in [0,1]$). Severe hospitalized individuals transmit the disease with infection rate β whereas mild/asymptomatic cases transmit the disease with infection rate $\phi\beta$. This difference in transmission is reasonable when assuming that, once hospitalized, individuals are

Parameters, variables and initial conditions	Description	Values
N	population size	2.2×10^6
$H(t_0)$	severe disease and hospitalized	54.0
$A(t_0)$	mild disease and asymptomatic	80.0
$U(t_0)$	ICU patients	10.0
$R(t_0)$	recovered	1.0
$C_H(t_0)$	recorded $H(t_0)$	54.0
$C_A(t_0)$	recorded $A(t_0)$	40.0
$C_U(t_0)$	recorded $U(t_0)$	10.0
$C_R(t_0)$	recorded $R(t_0)$	1.0
$D(t_0)$	death	1.0
β	infection rate	$3.25 \cdot \gamma$
ϕ	ratio of mild/asymptomatic infections contributing to force of infection	$1.6[1.0 - 2.0]$
γ	recovery rate	$0.05d^{-1}$
μ	disease induced death rate	$0.025d^{-1}[0.02 - 0.03d^{-1}]$
ν	hospitalized to ICU rate	$0.025d^{-1}[0.025 - 0.1d^{-1}]$
η	proportion of hospitalization	$0.45[0.0 - 0.5]$
ξ	detection ratio of mild/asymptomatic	$0.45[0.0 - 0.8]$
ρ	import parameter	$-$

Table 1: Model parameters and initial condition values.

isolated and would no longer transmit as much as mild/asymptomatic cases, often undetected and more mobile. Infected individuals may recover with a recovery rate γ, however, severe hospitalized individuals could be also admitted to the ICU facilities, with a rate ν, or die before being admitted to the ICU facilities, with disease induced death rate μ. Individuals admitted to the ICU facility could eventually recover with a recovery rate γ or die with disease induced death rate μ.

The model is calibrated using the empirical data for the Basque Country and the biological parameters are estimated and fixed as the model is able to describe the disease incidence during the exponential phase of the epidemic for each dynamical class. Parameter insecurities are quantified with likelihood functions. Model parameters and initial conditions are shown in Table 1, where β is the infection rate and ϕ is the ratio describing the asymptomatic/mild infections contribution to the force of infection. γ is the recovery rate, μ is the disease induced death rate and ν is the rate of hospitalized going to the ICU. η is the proportion of susceptible being infected, develop sever symptoms and being hospitalized whereas $1-\eta$ is the proportion of susceptible becoming infected and developing mild disease or asymptomatic. ξ is the ratio of detected, via testing, mild/asymptomatic infect individuals. ϱ is the import rate needed

to describe the introductory phase of the epidemics and for the present study, we assume ϱ to be much smaller than the other additive terms of the force of infection, given the strong observational insecurities on the data collected at the beginning of the outbreak.

1.1 The refined model

The original SHARUCD model was refined to synchronize ICU admissions to hospitalizations and positive tested infected, rather than to deceased and recovered. We consider primarily SHARUCD model versions as stochastic processes in order to compare with the available data which are often noisy and to include population fluctuations, since at times we have relatively low numbers of infected in the various classes.

We change the transition into ICU admissions from the form like used in recovery γ and death μ, more to the one for distinction of hospitalized and asymptomatics η, to describe the results from the growth rate analysis of all data sets, see previous subsection. Hence we update the transitions just a bit from

$$\begin{aligned} w_1(\underline{x}) &= \eta\beta x_1(x_2 + \phi x_3 + \varrho) \quad , \quad \underline{r}_1 = (1, -1, 0, 0, 0, -1, 0, 0, 0, 0)^{tr} \\ w_7(\underline{x}) &= \nu x_2 \quad , \quad \underline{r}_7 = (0, 1, 0, 0, -1, 0, 0, -1, 0, 0)^{tr} \end{aligned} \tag{9}$$

into

$$\begin{aligned} w_1(\underline{x}) &= \eta(1-\nu)\beta x_1(x_2 + \phi x_3 + \varrho) \quad , \quad \underline{r}_1 = (1, -1, 0, 0, 0, -1, 0, 0, 0, 0)^{tr} \\ w_7(\underline{x}) &= \eta\nu\beta x_1(x_2 + \phi x_3 + \varrho) \quad , \quad \underline{r}_7 = (1, 0, 0, 0, -1, -1, 0, -1, 0, 0)^{tr} \end{aligned} \tag{10}$$

and have to adjust the parameter ν, which was an ICU-admission rate in units of d^{-1} into an ICU-admission ratio $\nu \in [0, 1]$. Parameters were slighly adjusted and for the refined model we use $\nu = 0.1$, $\mu = 0.02$, $\phi = 1.65$, $\xi = 0.4$ and $\eta = 0.4$ and obtain immediately a very good agreement now of ICU data and simulations, with the empirical data for each variable lying in the median range of stochastic realizations, and only small deviations in the other variables. The other parameters values were kept the same as shown in Table 1.

2 Two parameter likelihood plots

We analyzed possible correlations between parameters by inspecting numerical two-parameter likelihood functions. Since the growth factor λ is mainly determined as $\lambda \approx \beta - \gamma$ we observe in

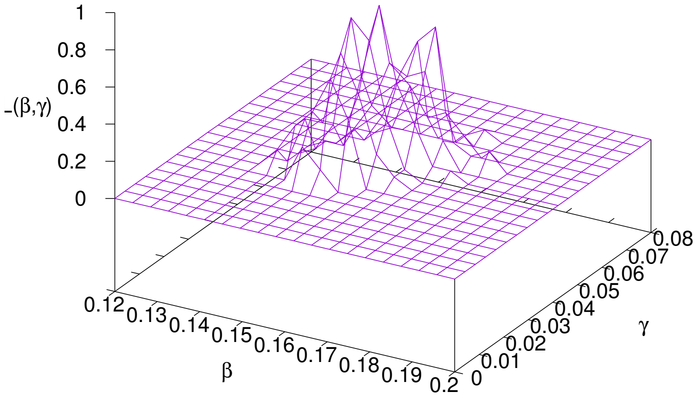

Figure S1: Two-parameter likelihood plot $L(\beta, \gamma)$ for parameters the infection rate β and the recovery rate γ, showing fewer than expected correlations between these two parameters which essentially determin the common growth factor λ_1. This well determined range of possible values for the parameters β and γ might be due to the available information from all 5 data sets, including the recovered in good quality.

many basic epidemiological models large correlations between especially these two parameters. Large infectivity β and small recovery period γ^{-1} describe often data as well as small infectivity and long recovery period. However we observe in a first inspection of the numerical likelihood of these two parameters, obtained from considering all 5 paremter sets in comparison with the SHARUCD basic model, that the range of possible values for β and γ together, see in Fig. S1 the area lifted away from zero in $L(\beta, \gamma)$, is quite restricted around the best values for each of the parameters individually, as they are shown in Fig. 3 a) and b) in the main text.

Furthermore, combinations between the parameters describing the distiction between severe and mild infecteds could be expected to show large insecurities in the individual parameters as well as large correlations between combinations of them. Hence we investigate in numerical two parameter plots the combinations of the severity ratio η with the infectivity ratio of mild infected as compared to the severely diseased, the ratio ϕ, hence $L(\eta, \phi)$, and the detection ratio ξ of mild or asymptomatic, hence $L(\eta, \xi)$, and further the likelihood of the two last mentioned parameters $L(\phi, \xi)$, see Fig. S2.

The likelihood $L(\eta, \phi)$, Fig. S2 a), is well in a range close to what we would expect form the individual likelihood plots in Fig. 3 e) and f), main text. The largest correlations are visible in the plot for $L(\eta, \xi)$, Fig. S2 b), where we observe non-vanishing probabilities of parameters

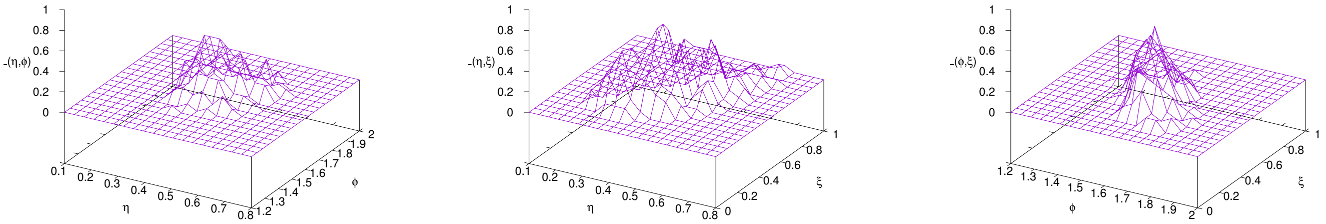

a) b) c)

Figure S2: Two-parameter likelihood plot a) for $L(\eta, \phi)$, b) for $L(\eta, \xi)$, and c) for $L(\phi, \xi)$, the parameters which describe more internal effects of hospitalization ratio η versus asymptomatic/mild, and infectivity of mild/asymptomatic ϕ, and detection rate of mild/asymptmatic ξ. Largest correlations between parameters are observed between η and ξ, see Fig. part b).

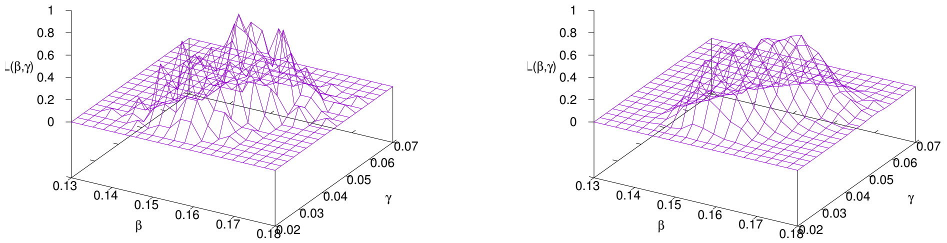

a) b)

Figure S3: Two-parameter likelihood plots for $L(\beta, \gamma)$ zooming into the parameter regions of β and γ, in a) comparing ensembles from the master equation simulations via Gillespie algorithm with the empirical data, and in b) using ensembles from the Fokker-Planck approximation simulating the stochastic differential equation system. The elevated areas of both graphs show a similar parameter region, confirming that the original approach a) already gives good informations about the two-parameter likelihood, and refinements in b) given a smooth likelihood surface, since larger ensembles are now better accessible.

for small hospitalization rate η and small detection rate of mild/asymptomatic infections ξ as well as for large η and large ξ. However, the severity rate η is well restricted between 0.2 and 0.7 and a clear maximum visible around the best value from the individual likelihood plot, Fig. 3 e) in the main text. The boundary value of $\xi = 1$ has a non-vanishing probability to explain the data, but numerically the value of L there is much smaller than the values in the middle of the parameter intervals of η and ξ. Hence, even with higher numerical resolution, on the expense of many more stochastic runs of the model, it is not expected to have high values of $L(\eta, \xi)$ still at locations close to the bundaries of the possible parameter values. So there are considerable correlations between these two parametes detected, but they seem to be mild, and from the available data we can obtain good information also about the most likely values of these parameters. Finally, for the parameter combination of $L(\phi, \xi)$ we observe again a more restricted area of possible parameter sets, though also here some correlations are visible. In conclusion, it seems that the available data sets give quite some information about the possible parameter combinations to describe the system under investigation. Further refinements of these analyses might give further insight into the dynamics of the epidemic.

Due to initially small numbers of infected, hospitalized cases etc., we use initially the Gillespie algorithm for the state discrete Markov process described by the master equation [2, 3]. The present two parameter likelihood plots become occasionally very computationally demanding, depending on system size N and numbers of transitions, especially for larger infectivity. hence the well tested approximation via the Fokker-Planck equation as stochastic differential equation system [4] speeds up the simulations, allowing for smoother likelihoods, but occasionally might give some errors in the small number situations. A carefull monitoring is therefor needed, see a first comparison of master equation likelihoods, using Eq. 3 from the main text, with stochastic differential equation likelihoods, using Eq. (6), with encouraging results Fig. S3, but need to be further investigated in future studies. However, the first results given here in Figs. S1 and S2 are already informative for the purpose of detecting eventual correlations between parameters, and encouraging for further intensive studies. The up to now detected correlations keep well within the expected ranges indicated from the one parameter numerical likelihood plots in Fig. 3 in the main text.

References

[1] Allen, E.J., Allen L.J.S, Arciniega, A., Greenwood, P. Construction of equivalent stochastic differential equation models. Stochastic Analysis and Application 26, 274-297 (2008).

[2] Gillespie, D.T. A general method for numerically simulating the stochastic time evolution of coupled chemical reactions. Journal of Computational Physics 22, 403-434 (1976).

[3] Stollenwerk, N. and Jansen, V. Population Biology and Criticality: From critical birth–death processes to self-organized criticality in mutation pathogen systems, Imperial College Press, World Scientific, London, 2011).

[4] Stollenwerk, N. et al. Hopf and torus bifurcations, torus destruction and chaos in population biology. Ecological Complexity 30, 91-99 (2017).
